# House modifications as a malaria control tool: how does local context shape participants’ experience and interpretation in Uganda?

**DOI:** 10.1186/s12936-023-04669-1

**Published:** 2023-08-25

**Authors:** Miriam Kayendeke, Christine Nabirye, Susan Nayiga, Nelli Westercamp, Samuel Gonahasa, Agaba Katureebe, Moses R. Kamya, Sarah G. Staedke, Eleanor Hutchinson

**Affiliations:** 1 Infectious Diseases Research Collaboration, Kampala, Uganda; 2 Malaria Branch, Centers for Disease Control and Prevention, Atlanta, USA; 3 London School of Hygiene and Tropical Medicine, London, UK; 4 Department of Medicine, Makerere University, Kampala, Uganda

**Keywords:** Malaria, House modification, Full screening, Partial screening, Eave tubes, Eave ribbons, Local context

## Abstract

**Background:**

Evidence that house design can provide protection from malaria is growing. Housing modifications such as screening windows, doors, and ceilings, and attaching insecticide-impregnated materials to the eaves (the gap between the top of the wall and bottom of the roof), can protect against malaria. To be effective at scale, however, these modifications must be adopted by household residents. There is evidence that housing modifications can be acceptable, but in-depth knowledge on the experiences and interpretation of modifications is lacking. This qualitative study was carried out to provide a holistic account of the relationship between experiences and interpretations of four types of piloted housing modifications and the local context in Jinja, Uganda.

**Methods:**

Qualitative research was conducted between January to June 2021, before and during the installation of four types of housing modifications. The methods included nine weeks of participant observations in two study villages, nine focus group discussions with primary caregivers and heads of households (11–12 participants each), and nine key informant interviews with stakeholders and study team members.

**Results:**

Most residents supported the modifications. Experiences and interpretation of the housing modifications were shaped by the different types of housing in the area and the processes through which residents finished their houses, local forms of land and property ownership, and cultural and spiritual beliefs about houses.

**Conclusions:**

To maximize the uptake and benefit of housing modifications against malaria, programme development needs to take local context into account. Forms of local land and house ownership, preferences, the social significance of housing types, and religious and spiritual ideas shape the responses to housing modifications in Jinja. These factors may be important in other setting.

*Trial registration* Trial registration number is NCT04622241. The first draft was posted on November 9th 2020.

## Background

Malaria remains a major health challenge across the globe contributing to over 500,000 deaths in 2020 [1]. As in many countries in Africa, houses are a high-risk space for malaria transmission in Uganda as most malaria vectors feed at night [2, 3]. Basic features which prevent mosquitoes from entering houses appear to provide protection against malaria infection [4–7]. Houses with screened windows, eves and ceilings have fewer mosquitoes inside and reduce the risk of being bitten by mosquitoes for people living there. People who live in screened houses (screens windows, doors, even and ceilings) have a 32% reduction in malaria parasite prevalence [8] compared those who live in houses without these features. Most houses in malaria-endemic areas do not have these features therefore there is need for them to be added to enable a reduction in mosquito density and reduce the risk of acquiring the malaria parasite for the people who live there.

With promising results from intervention trials, strategies should be developed to support the uptake of and adherence to housing modifications. Acceptability studies are an important first step in understanding community responses to interventions. Studies have shown that screening (doors, windows, or ceilings) in the Gambia [5, 9–12] or installing eave ribbons in houses in Tanzania [13] are acceptable approaches. Window and door screens (and in The Gambia, ceiling screens) are valued for limiting entry of mosquitoes, insects, and dirt into houses, and are thought to improve privacy [5, 9–12]. In The Gambia screening also stopped animals from entering homes, were said to look beautiful, and improve security [9, 10]. Studies have, however, produced mixed reports on the effect of screening on the temperature inside houses [14, 15]. In The Gambia, people complained that screens could be damaged by small children [10], were hard to clean and, once damaged or if poorly constructed, could look untidy [9].

While important, the policy relevance of these studies is limited. They rely on definitions of acceptability created by researchers or programme managers rather than the recipients themselves and rarely go beyond an interest in the physical properties of the modifications [16]. The successful introduction of house screening requires local shifts in construction practice and changes in decision-making by individuals and families on how to invest scarce resources. For the achievement of equitable coverage, some household residents will have to agree to their implementation through top-down intervention [8]. Decision-makers wishing to form effective policy will need a more detailed, bottom-up account of how local social, political, and economic context is likely to support, or limit, the introduction of housing interventions.

Holistic, ethnographic accounts of the experiences and interpretations of the recipients of housing modifications can provide these critical contextual maps for policy makers. This paper provides an account of a pilot study, which was conducted to assess the feasibility of introducing four types of housing modifications to combat malaria from the perspective of household residents. Drawing on focus group discussions (FGDs), ethnographic observations, and key informant interviews, it explores how forms of land ownership, challenges and changes in the local economy, and cultural and spiritual practices shaped the experience and interpretation of housing modifications in three villages in Jinja, Uganda.

## Theory, context, and methods

Globally, Uganda is the third highest contributor to malaria cases and accounts for 5% of all infections across the world [17]. Although major progress has been made in reducing malaria burden in the country in the last 20 years, the national mean monthly incidence rate of malaria remains 20.4 cases per 1000 [18]. This study was conducted in Jinja district. It is part of the Busoga region which has one of the highest incidence rates of malaria in the country, reported to be 73.1 per 1000 in some districts during peak months [18]. Jinja district is an area of perennial malaria transmission in Eastern Uganda [19]. Ethnically, the main inhabitants of the district are Basoga, but the relatively vibrant local economy has drawn members of many other ethnic groups, including Baganda and Luo [20, 21].

The pilot study that we followed was conducted in the predominantly rural part of the district. This study area was selected basing because of the high burden of malaria and ongoing challenges with pyrethroid resistance, the willingness of the local leadership to support the study and availability of infrastructure to enable the team to evaluate the intervention through the health facilities.

Subsistence farming and cash cropping dominates the economy. Across the district, sugarcane has been the major cash crop since colonial times, becoming a primary source of household income through a private ‘out grower’ scheme to supply sugarcane to local factories [21]. In recent years, sugarcane prices have fallen and indebtedness to the main sugar producer makes it difficult for farmers to turn to more profitable produce [21, 22]. In this context, small informal enterprises are important to the local economy, but provide little profit and rarely draw people tout of poverty [23]. During 2020–2021, the prolonged impact of the COVID-19 pandemic and restrictions on the economy further escalated the difficult financial situation within households [24].

**Fig. 1:**
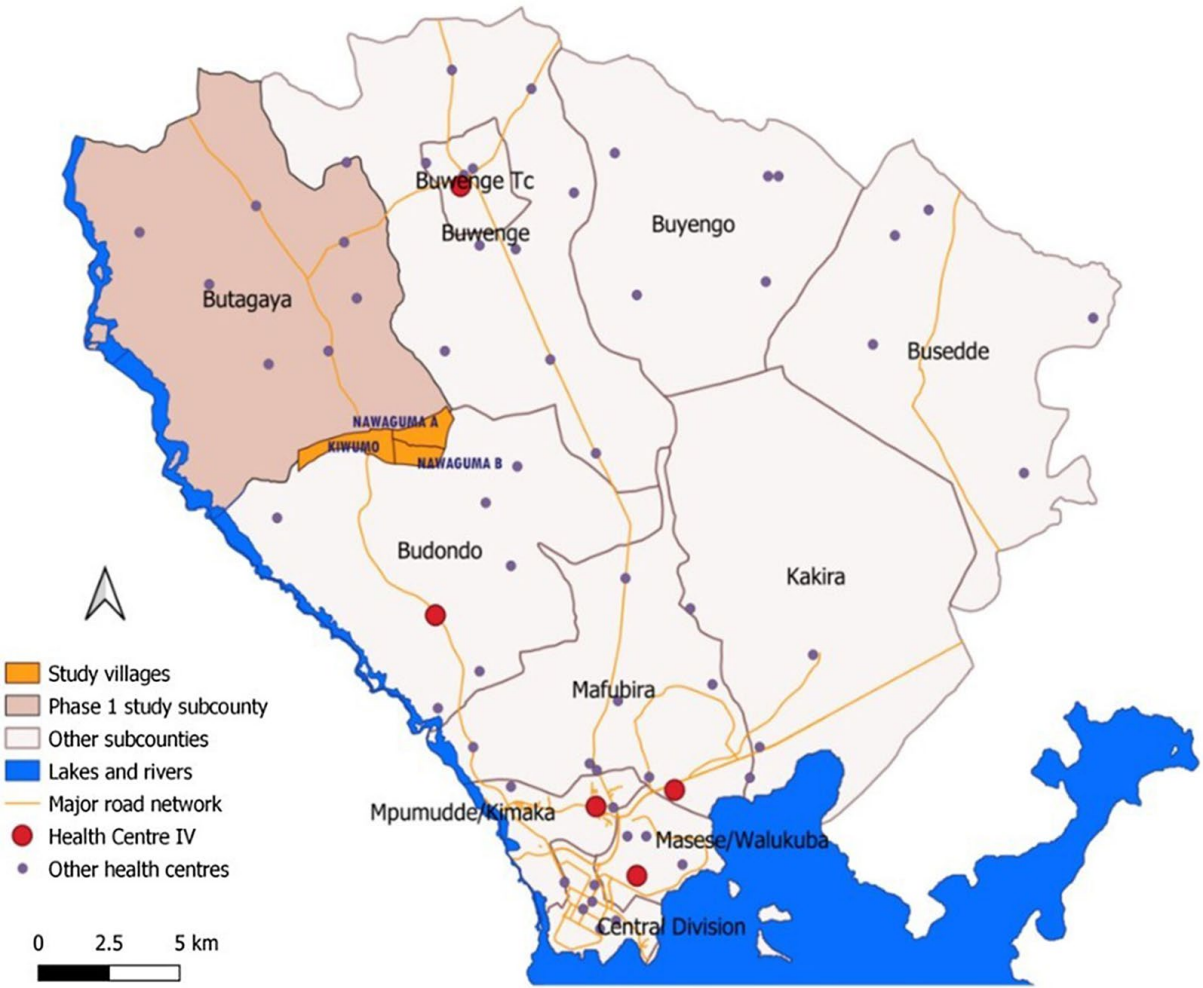
Map of Jinja district showing Butagaya subcounty pilot study site and the pilot villages highlighted

The study was to be conducted in two phases: a pilot (Phase I) and a full cluster randomized intervention trial (Phase II). The aim of the pilot was to develop and test four types of housing modifications in both modern houses (those with brick or stone walls) and traditionally constructed houses (those with mud walls). The two housing interventions which were most successful in the pilot study (feasible, acceptable, and effective) would then be selected for the full intervention trial. The housing modifications that were piloted included: (1) full house screening (eaves/ceilings, ventilation bricks/openings, and windows), (2) partial house screening (eaves or ceiling), (3) eave tubes, and (4) eave ribbons. All households had access to piperonyl butoxide (PBO) long-lasting insecticidal nets (LLINs) and all housing modifications were provided free of charge to all recipients. Three villages located in Butagaya sub-county, Jinja, were identified as they had a good mix of modern and traditional houses, fewer rented homes, and a willingness of local leaders to support the study (Fig. 1).

A total of 200 houses were enrolled in the pilot study. The houses were mapped using a handheld GPS device, and consent was sought from household members. The inclusion criteria were: (1) at least one adult aged 18 years or older present; (2) agreement of the adult resident to provide informed consent for the pilot study. Individual households were stratified into two categories (modern vs. traditional construction) with 100 households of each category type. The study used block randomization to assign 20 households from each category assigned to the 5 study arms (4 arms received 1 intervention listed in Table 1 plus PBO LLIN each; and 1 arm received PBO LLINs only). Modifications were implemented by local builders, carpenters, masons and potters who were selected from the study villages upon recommendation by the local leaders and who got trained and managed by a project engineer during February and March 2021. Project activities were funded by the U.S. President’s Malaria Initiative through the Infectious Diseases Research Collaboration (IDRC), a local research organization that coordinated the study activities. IDRC set up a study team whose role was to sensitize the study community leadership on study procedures, select households, consent household heads, guide the implementing team and ensure that all study activities were done in accordance with the study protocol and standard operating procedure. A small workshop was set-up in the study area where all the fabrications were done before installation were conducted at individual households. The consenting process, which was conducted by the study team engaged the household head of a selected household by reading the consent form in the local language. The process started by introducing the study title, leadership and funders. This was followed by reading detailed paragraphs on why the study was being done, why the household was selected, what their role will be in the study, a detailed explanation on the type of modification they will obtain and how it will be done, an explanation on the role of the project in providing this intervention, a discussion on risks and benefits to taking part in this study, a discussion on the participants’ rights to taking part in the study and finally a written consent to taking part in the study. The feasibility and effectiveness of the household modifications was assessed through a qualitative study, evaluation of the costs and implementation of the interventions, and entomology surveys (using CDC light traps). One to two housing interventions were selected for Phase II following the review and discussion of the pilot results with the trial steering committee. The recipients received the all the interventions in the pilot and the subsequent main trial for free.

**Table 1:** Types of modifications introduced during the project

Modification name	Description
Full house screening	Permanently fixing wooden frames with wire mesh to windows and air vents in the house either externally or internally (depending on the direction of the window openings); screening the eaves or installing screened ceilings if no ceiling was present, and sealing any open gaps/holes in the walls.
Partial house screening	Screening the eaves or installing a screened ceiling, where no ceiling was present.
Eave tubes	Installation of short PVC tubes, 15 cm in diameter, containing an insert with insecticide impregnated netting into the wall or behind existing ventilation holes.
Eave Ribbons	Installation of 1–2 m lengths of 15 cm-wide triple-layered hessian fabric strips treated with a mosquito repellent (transfluthrin) to houses around the eave spaces, without completely closing eave spaces.

Phase II will include a cluster-randomized trial comparing 2 interventions against a control arm across 60 clusters of 100 households. All households will receive PBO LLINs. The impact of the interventions will be assessed through a cohort study, cross-sectional community surveys, entomology surveillance, a qualitative study, and an economic evaluation. The primary outcome of the trial will be clinical malaria incidence in children aged < 60 months as measured in the cohort study. All housing modifications will be provided free of charge to all recipients.

### Qualitative methods and analyses

The overall objective of the qualitative study was to analyse local experiences and interpretations of the four housing modification types, and to understand the elements of the local context that shaped acceptability, as well as concerns about, or rejection of, the interventions among households.

The qualitative study comprised ethnographic observations, focus group discussions, and key informant interviews. The analysis took a poststructuralist approach and were interested in the interconnections that emerged between the study components and the local context. The housing modifications were conceptualized as ‘assemblages’, and explored how the practices, logics, and material resources embedded in the pilot study interacted with and were re-interpreted within the local context [25].

The qualitative team was separated from the main trial team in terms of day-to-day activities and supervision. The researchers collecting qualitative data were never involved in collecting trial data nor sensitizing or mobilizing the community. All qualitative researchers were mentored by an anthropologist based in the UK, who supported decision making around data collection; writing fieldnotes; analysing and interpreting data; and writing up.

### Ethnographic observations

Participant observation began five weeks before the first modifications were made and continued for four weeks during the installation, weekly for 3 days. Informal discussions held during this time explored the processes through which houses were built, lived in and destroyed, and sought a holistic understanding of meanings and roles that housing played in daily life within the study area [26]. The observations of the installations were aimed at understanding how the experiences of receiving modifications intersected with these experiences and interpretations.

Village health team members supported fieldworkers by identifying community members and local builders who were willing to participate in the ethnographic work. Discussions and informal interviews were recorded manually in notebooks as field notes. The field notes were typed, and their significance was discussed by the qualitative study team at the end of each week.

### Focus group discussions

Focus group discussions (FGDs) were conducted with residents at baseline (before installations) and one month after the installations. At baseline (see Table 2), four FGDs were conducted with 47 primary care givers and household heads from modern and traditional houses from two villages. One month following the installation, five FGDs were conducted with 60 residents from all three villages including primary care givers and household heads from modern and traditional houses as well as local labourers that were involved in installing the housing modifications. An FGD was held for each of the modification types and participants were selected using convenience sampling. Heads of households were included as they make decisions about household modifications. Care-givers were included as a group as the project was particularly concerned about infection among children, and wished to know if the intervention was supported among those who provide most childcare. The participant observation was not limited to household heads and caregivers. All FGDs were conducted in the local language (Lusoga) and translated into English using meaning-based translation [27].

**Table 2:** Sampling and populations for FGDs

Baseline (two villages)	Modern houses	Traditional houses
Heads of household (mix of male and female)	1 FGD (12 participants)	1 FGD (12 participants)
Primary caregivers (female only)	1 FGD (12 participants)	1 FGD (11 participants)
One month after installations (three villages)	Modern and traditional houses	
Full screening (mix of male and female)	1 FGD (12 participants)	
Partial screening (mix of male and female)	1 FGD (12 participants)	
Eave tubes (mix of male and female)	1 FGD (12 participants)	
Eave ribbons (mix of male and female)	1 FGD (12 participants)	
Local carpenters and masons (male)	1 FGD (12 participants)	

### Key informant interviews

A total of 9 key informant interviews were conducted: four with male key stakeholders (local leaders, health inspectors, opinion leaders) and with five male study team members who were involved in implementing the pilot study.

Interviews with key stakeholders were aimed at reflecting on the experiences of the modifications. The interviews with the pilot study team members were aimed at understanding how the interventions were received by the local community and any concerns that arose as well as their interpretation of the feasibility of installing the modifications. The interviews were administered using topic guides and recorded using a digital voice recorder. Contact summaries of the interviews were written after each interview and discussed by the qualitative team to identify any new emerging issues for exploration in subsequent interviews.

### Data analysis

All the data (field notes, transcripts from FGDs and IDIs) were uploaded and analysed in NVivo, QSR International Version 12. A coding scheme was developed by the team during data collection and themes and sub themes were identified. Field notes and transcripts from the interviews were read several times and ideas pertinent to the pre-determined themes and sub themes were derived and assigned to a category, a ‘code’; patterns seen amongst the ideas were grouped together and a coding structure developed. The coding structure was reviewed and discussed during weekly team meetings to reach a consensus. Two coders coded the data independently and created additional codes inductively. Overall, the data presented here are from 9 weeks of participant observations, 9 FGDs and 9 key informant interviews.

## Results

Residents often described that the modifications upgraded the quality and aesthetic appeal of their houses. The full screening modification, which represented the most substantial modifications to the houses, was by far the most preferred intervention. It was thought to instantly improve the look of the houses; the aluminum screens on the wooden window frames, and the white netting material that was used to screen the ceiling, were considered particularly aesthetically pleasing. Many participants were conscious that the screens protected them against mosquitoes, other insects, small animals (e.g., rodents), and debris falling from the roof. A resident who received the full screening intervention described her experience with the modification as follows:

*“After the installation, I felt peace when I saw the screens. Since they were placed outside of my window, whenever a person would pass by, they would say ‘As this one looks good!’ ‘As they are beautiful!’ so it changed the look of my house”. (R8, FGD with participants that received full house screening)*

Partial screening was initially well-received by residents because the white netted material that was installed as an indoor ceiling was considered attractive. There were initial reports that the netted ceilings reduced exposure to mosquitoes, rodents, and heat from the iron sheets, by serving as a barrier for debris and insects, which fell from the roof. However, many residents complained that rodents chewed holes in the netted ceiling, limiting their durability. As a result, the netted ceilings became less popular over time, and when prompted to see if participants would prefer this method, many were skeptical about investing in this form of modification.

*“The work of the net of the ceiling spoke for itself 100%. Before installation, the entomology team trapped 7 mosquitoes, this time when they came there wasn’t a single mosquito. So there is a change in the mosquitoes up there on the net. However, rats are disturbing us! They come and bite the ceiling thereby creating holes and taking away security. They bite it. The net is weak!” (R6, FGD with residents that received partial screening)*.

The eave tube modifications were also less popular among residents. Some residents disliked their round shape, and for some, placing the eave tubes on plywood behind the ventilation bricks was odd. Owners of both traditional and modern houses were concerned about the installation process, fearing that electric drilling would damage the walls of their houses (although this concern was overcome when the team modified the approach to installation).

*“Some people refusing say, ‘for me at mine don’t come and do this,’ but later they agreed. But mostly those who were refusing are those with brick (modern) houses which are well built. The eave tube is the type that they feared most. They were saying ‘if you modify my house it will get spoilt’.” (P6, FGD with local labourers)*

Eave ribbons were the least popular intervention among the recipients. They complained that the hessian fabric material that was hung around the eave spaces—between the face board and wall of the house—was a simplistic intervention, an inferior modification that was unattractive. Over time, however, as residents noticed that the eave ribbons deterred mosquitoes and other insects, they appeared to gain favour.

Overall, residents were positive about all four types of modification, which was supported by the fact that no household refused to be involved in the study. There were, however, three elements of the context which shaped the experience of the modifications and had to be managed by the project. These are explored below.

### Context I: housing modification and fear of land being grabbed

In the study area, land and housing was a major source of security and a sign of independence for many residents, which shaped how the housing interventions were initially viewed and received. Many residents described how they owned land through customary land tenure, which is inherited through patrilineal or patrilocal ties. In many households, when boys reached puberty, they would be provided a piece of land on which to construct a house as part of their pathway to maturity and independence. This land is inherited through the male child or through women residing in their husband’s villages. While these patterns of land ownership were still evident in the district, as more urban settlements have emerged, land was often bought and sold after being split into smaller plots. The recent changes in status of the study area into a town council, and the nearby Jinja town into a ‘city’ meant that land prices were increasing, and new forms of local development were created to cater for newcomers to the area.

The increase of prices coupled with the poor legal protection that customary land tenure provides in Uganda [28] left many residents who occupied land along customary arrangements concerned that new rules for urban planning would lead to land grabbing. In this context, land grabbing mainly refers to the intimidation of communities to abandon or be forcefully removed from their land for agricultural or commercial expansion. There were rumours that escalated the fear of loss of land among residents that a piece of land on the outskirts of the city was to be transformed into a waste site servicing Jinja City and that those without written proof of ownership would have their land taken. Further, concerns were expressed among some residents that houses constructed without approved plans from the town council authorities would be destroyed.

In this context, when the study team took photographs and recorded GPS coordinates of houses for enumeration purposes, residents were worried and many interpreted these actions as a plan to grab land, as expressed by a local leader in the quote below.

*“At first people were worried that their land was going to be taken. [At] that time when the project had just started and coordinates were being picked, there was fear. Propaganda… because of the coming of the city [there was fear that] that their land is going to be taken or stolen, so that fear was there”. (IDI with the Health Assistant)*

In these situations, the involvement of the local community leaders was critical in liaising between the project and local residents. Leaders explained the project activities to the residents, and enabled community concerns to be conveyed to local leaders and then to the study team at routine sensitization meetings. For example,

*“After explaining to them the importance and purpose of the study, they accepted… But instead, others [who did not receive the intervention] were not happy because they did not get that chance [to modify their houses]”. (Interview with the Health Assistant)*

The involvement of local leaders helped curb rumours about land grabbing, but participants also took steps to protect their properties. Traditionally, land in the area is owned by men and women do not, therefore, have the legal right to sell. When consent was being sought for the modifications to be made, some male household heads insisted that their wives living in the house sign consent forms thus protecting themselves from any attempt to grab their land.

Concerns about trustworthiness of the project also shaped the relationship between the builders installing the modifications and the residents. Even though the project had specifically chosen to use local builders aiming to foster local ownership and improve relationships between the project and local community, community residents raised concerns that builders were entering private spaces that were rarely seen by those outside the household. Residents were worried that builders might steal their property, but also that they might gossip about the socio-economic status of those living in the house. As one FGD participant described:

*“For us we shall not accept them exposing us! When they come to put the net [screen] in the house, you allow them to move [all over] the whole house. They even talked about me …they said, ‘that one is not badly off’. They grade, those men are gangsters they are impossible! The other thing they [residents] complained about was that the builders are thieves. There’s a lady whose money they stole and there’s another they stole a memory card from and even that habit of announcing, there’s a person they exposed that she sleeps on a papyrus mat. The builders were the ones talking. Then their wives started spreading that news, that she does not have a proper place to sleep”. (P 11, FGD with residents that had obtained partial screening)*

While local leaders were pleased with the employment opportunities and potential for sustainability the project provided, using local labor to make changes in households cannot be assumed to bring trust and ownership into the project. When concerns about the trustworthiness of the builders was raised with the project team, they responded by retraining the labourers, emphasizing the need to maintain the privacy and confidentiality of the participants.

## Context II: poverty, housing types and unfinished dwellings

The intensification of land clearance to grow sugar cane impacted on housing in the local area in two ways. First, it reduced the availability and increased the cost of grass and tree poles to construct traditional houses, and second, the recent reduction in income from sugar cane production meant that many of the more prestigious, baked brick houses were left unfinished when residents failed to realize expected prices for their crops.

Throughout the area, local leaders and residents described how mud brick with a thatched or corrugated iron roof continued to be a cheaper option for residents wishing to build a home, and this remained the case despite the difficulty of accessing materials locally. Study participants often described them as inferior, transitional, or temporary structures, and most residents of these houses aspired to build permanent, baked brick European style bungalows with separate rooms, including a sitting room, bedrooms, kitchen, bathrooms, and a garage, with large windows to allow sunlight in. The high status of these baked brick houses is reflected in their name *‘bugaga bukomye’* which translates literally as *‘epitome of wealth*’, a means of celebrating the substantial amount of money that has been spent on the house. Residents preferred the larger *‘bugaga bukomye’* which they believed would provide better ventilation, and included a ceiling, which reduced heat inside the house. Once plastered and painted, these houses were considered very attractive and raised the status of the residents within the community.

Few residents, however, had sufficient funds to complete construction of such houses all at once; building continued step-by-step when funds were available. As a result, most owners were forced to live in unfinished dwellings, becoming resident once the roof, doors and window spaces were in place. Many houses with building ‘in progress’ had no ceilings, unplastered walls, unfinished floors, and metal roofing sheets which were weighted down with bricks or timber, rather than attached to the walls. Construction holes that enabled scaffolding to be attached to the house were often left unfilled so that building work could continue, and with no money to purchase windows, open spaces were either left fully open or covered with various materials, including plywood, iron sheets, or unbaked bricks, instead of glass.

In this context, where many residents could not afford to complete their ‘modern’ houses, the house modifications were welcomed. They were considered by the local residents to be a substantial investment, which they would have been unable to make themselves. While those living in mud-brick houses often welcomed the intervention, they also expressed surprise that improvements were to be made in what they saw as temporary dwellings. This was especially true regarding eave tubes, which were sometimes described as ‘modern ventilators’ by participants.

### Context III: housing modifications, spirits, and cultural practices

Many of the people living in the villages in the study area identified themselves as very spiritual and spoke about a powerful charismatic traditional healer whose daughter continued to be an important, well-known healer among the residents. Spirits were thought to reside with people in their houses and while they could live happily with them, these spirits could also be a threat to the health of those who lived there. One house which was enrolled into the pilot, was pulled down by the family living there shortly before the intervention began. The house had been built by a young man who, according to customary land tenure, had been given land on the compound owned by his family when he had become a youth. Following his divorce from his first wife and marriage to a second woman, rumors circulated that his first wife had in fact been a close cousin and that he had offended spirits through what is locally understood to be an act of incest. The deaths of two young children during the man’s second marriage, led the couple to abandon the house and leave the village. Even though the family was keen to be part of the study, concerns that malevolent spirits inhabited the house led to its destruction, and their withdrawal from the project.

While only one house that was explicitly withdrawn from the project because of concerns about spirits, many householders were worried about the potential to block what was known locally as ‘spirit ventilation’. In both modern and traditional houses where twin children either lived or had lived when they were young, ventilation holes were created in the parents’ bedrooms on the back wall which ranged from larger holes (approximately 10 cm^2^) to those that were invisible to the naked eye.

Accompanying these holes could be a set of sticks demarcating the site, a bowl placed strategically within that little house to accommodate daily offerings and sometimes a basket containing the umbilical cords of the twins—wrapped in cloth with cowrie shells. As described by several informants and in the anthropological literature on Uganda [29], these practices stem from the idea that twin children are highly spiritual but also at risk from potentially malevolent spirits who enter the house when twin children are born. These spirits can murder the twins, but only if the spirits became trapped in the house; the holes and the additional objects ensure that the twins’ spirits come and go freely. Even though many builders were from the local area, these holes were often blocked as part of the modifications, surprising the residents.

*“When they go to build [install modifications], they should tell us because you can find a hole for the twins and cover it up without knowing so the builders end up getting in trouble.” (P8, FGD with residents of households that received eave tubes)*

Spiritual beliefs related to having twins is an example of cultural considerations that must be understood before modifying houses. Local beliefs surrounding spirit movement in certain houses (i.e., with twins, here) are essential to explore before implementation of such initiatives in the community.

## Discussion

Housing modifications are a promising new strategy against malaria. Studies suggest that screening windows, ceilings/eaves, and doors, and placing eave tubes and eave ribbons in houses is acceptable. There is, however, little discussion of the influence of the local context on participant experience and interpretation of the implementation of modifications in urban and rural settings. Knowing how the intervention intersects with different elements of society is useful and can provide a map or set of issues that should be considered when interventions are being scaled up.

This qualitative study was carried out to provide a holistic account of local interpretations and experiences of the four housing modification types in Jinja. The findings suggest that forms of land and property ownership, poverty and difficulties completing house construction, and ideas about the spirit world, shaped experiences and interpretation of the interventions as they were introduced to study residents.

Land and housing remain a major source of security and independence for the residents [30], serving as a source of power through inheritance and purchase, but also providing security. Customary land ownership of ancestral grounds in Busoga is highly valued to facilitate continuity of family lineages [30]. Land grabbing often occurs under the guise of development when local leaders conspire with those outside the district to sell valuable land and properties [31, 32]. There was widespread anxiety about the project within the community, especially at the beginning of the intervention when pictures and GPS coordinates were taken for each household. Some form of external assistance by the State, NGOs or donor agencies will likely be needed in order to achieve equitable distribution of housing modifications in countries like Uganda. This will likely involve mapping and taking GPS coordinates to identify suitable houses, which will have to be done carefully to avoid unnecessarily worrying members of those communities.

As public health actors become more involved in housing [7], they will need to be cognizant of the politics of land and development within a given area, to better understand how they may shape uptake of interventions, and to guide strategies to mitigate their impact.

As in other parts of the country, house design was changing from traditional mud-walled houses to modern baked brick bungalows [33]. In the study area, however, the steady drop in prices of sugar cane over the past 6 years [34] meant that many families struggled to complete construction of their houses, leaving many entry points for mosquitoes. The difficulties with finishing houses seem to have made the interventions attractive to those living in the area, but raise questions about equity—whether those who were most appreciative of the modifications would be least able to afford to put them into houses themselves. In addition, those living in traditionally constructed houses were surprised by the offer to install the modifications in their homes. Although families in our study area often live in these traditional houses for many years, such houses are described as temporary dwellings and, therefore, not worth investing in. As modification work continues, it will be important to modify these structures but strategies may need to be developed to convince people that these investments are worthwhile and protect the poorest segment of rural population that is also most vulnerable to malaria.

The importance of engaging local leaders to enable the successful implementation of public health interventions is well documented [35, 36]. Failure to acknowledge cultural practices, as well as local political and economic structures and tensions, can be catastrophic. Rumours about the ‘real’ intention of interventions, including vaccines, mass drug administration, malnutrition treatments, and emergency support, can derail effective interventions for poor and vulnerable groups [37, 38]. If those seeking to implement housing modifications to prevent malaria recognize and acknowledge the local political and economic environment, land ownership practices, how different types of housing are interpreted, and the ways that spiritual beliefs and cultural practices shape construction, then they can act to mitigate issues early on. These results suggest that local social and political leaders must be important partners, to ensure high uptake of housing interventions.

This study had several limitations. First, the qualitative assessment was carried out in only a few selected households in two villages. However, this approach provided an in-depth understanding of key contextual issues that need to be taken into consideration when implementing housing modification interventions in low resource settings. Extensive ethnographic observations enabled a better understanding of the housing context in the study area before and during the installations of interventions. Second, the qualitative team’s position as researchers and evaluators of the four housing modifications, and how the community members perceived the role of the study team, may have influenced what information household members and research participants were willing to share. Finally, in this article, the focus is on what is valued about housing and what housing represents in this setting to give insights on key factors that shape the acceptability of housing modifications to prevent malaria, rather than more broadly on overall health or other goals of housing construction.

## Conclusions

The experience and interpretation of housing modification for malaria prevention in this setting were shaped by the local socio-economic and cultural context in which they were embedded. Despite the initial concerns about land grabbing, the intervention was seen to upgrade houses and protect people from mosquitoes and rodents. This study recommends that future interventions and, if deemed appropriate in Uganda, the successful scaling up of modifications will need to take account the context in which such modifications will take place. This research suggests that knowledge on social relations, political-economy, and land; and the spiritual and cultural nature of housing prior to introducing any changes will enhance acceptance and the uptake of these promising new interventions to protect people from malaria.

## Data Availability

The qualitative data that support the findings of this study are not publicly available, as this would compromise participant privacy. Participants did not consent to have their interview transcripts made publicly available. However, data may be available from the corresponding author on reasonable request.

## Abbreviations

GPS: Global positioning system
LLIN: Long-lasting insecticidal nets
WHO: World Health Organization
